# Non-Standard Employment and Unemployment during the COVID-19 Crisis: Economic and Health Findings from a Six-Country Survey Study

**DOI:** 10.3390/ijerph19105865

**Published:** 2022-05-11

**Authors:** Virginia Gunn, Alejandra Vives, Alessandro Zaupa, Julio C. Hernando-Rodriguez, Mireia Julià, Signild Kvart, Wayne Lewchuk, Eva Padrosa, Mattias Philippe Vos, Emily Q. Ahonen, Sherry Baron, Kim Bosmans, Letitia Davis, Ignacio Díaz, Nuria Matilla-Santander, Carles Muntaner, Patricia O’Campo, Per-Olof Östergren, Christophe Vanroelen, Emilia F. Vignola, Theo Bodin

**Affiliations:** 1 Unit of Occupational Medicine, Institute of Environmental Medicine, Karolinska Institutet, 11365 Stockholm, Sweden; julio.hernando@ki.se (J.C.H.-R.); signild.kvart@ki.se (S.K.); nuria.matilla.santander@ki.se (N.M.-S.); theo.bodin@ki.se (T.B.); 2 MAP Centre for Urban Health Solutions, Li Ka Shing Knowledge Institute, Unity Health Toronto, Toronto, ON M5B 1W8, Canada; patricia.ocampo@unityhealth.to; 3 Lawrence S. Bloomberg Faculty of Nursing, St. George Campus, Toronto, ON M5T 1P8, Canada; carles.muntaner@utoronto.ca; 4 Department of Public Health, School of Medicine, Pontificia Universidad Católica de Chile, Santiago 8330077, Chile; alejandra.vives@uc.cl (A.V.); alessandro.zaupa@gmail.com (A.Z.); idiaz.concha@gmail.com (I.D.); 5 CEDEUS—Center for Sustainable Urban Development, Pontificia Universidad Católica de Chile, Santiago 7520246, Chile; 6 ESIMar (Mar Nursing School), Parc de Salut Mar, Universitat Pompeu Fabra-Affiliated, 08003 Barcelona, Spain; mireia.julia.perez@psmar.cat (M.J.); eva.padrosa.sayeras@psmar.cat (E.P.); 7 Social Determinants and Health Education Research Group (SDHEd), Hospital del Mar Medical Research Institute (IMIM), 08003 Barcelona, Spain; 8 Research Group on Health Inequalities, Environment, and Employment Conditions Network (GREDS-EMCONET), Department of Political and Social Sciences, Universitat Pompeu Fabra, 08005 Barcelona, Spain; 9 Department of Economics and School of Labour Studies, McMaster University, Hamilton, ON L8P4M9, Canada; lewchuk@mcaster.ca; 10 Interface Demography, Department of Sociology, Vrije Universiteit Brussel, Pleinlaan 2, 1050 Brussels, Belgium; mattias.philippe.vos@vub.be (M.P.V.); kim.bosmans@vub.be (K.B.); christophe.vanroelen@vub.be (C.V.); 11 Division of Occupational and Environmental Health, Department of Family and Preventive Medicine, University of Utah School of Medicine, Salt Lake City, UT 84108, USA; emily.ahonen@utah.edu; 12 Barry Commoner Center for Health and the Environment, Queens College, City University of New York, New York, NY 11367, USA; sherry.baron@qc.cuny.edu; 13 Independent Researcher, Boston, MA 02108, USA; lkdavis49@gmail.com; 14 Dalla Lana School of Public Health, University of Toronto, Toronto, ON M5T 1P8, Canada; 15 Department of Mental Health, The Johns Hopkins University Bloomberg School of Public Health, Baltimore, MA 21205, USA; 16 Social Medicine and Global Health, Department of Clinical Sciences Malmö, Lund University, 20205 Malmö, Sweden; per-olof.ostergren@med.lu.se; 17 Department of Community Health and Social Sciences, Graduate School of Public Health and Health Policy, City University of New York, New York, NY 10025, USA; emilia.vignola45@sphmail.cuny.edu; 18 Center for Occupational and Environmental Medicine, Stockholm Region, 11365 Stockholm, Sweden

**Keywords:** health equity, social inequalities, poverty, worker health and well-being, mental health, occupational health and safety, transitions towards non-standard employment and unemployment, income and employment insecurities, lack of workplace rights, pandemic responses and recovery strategies, atypical employment

## Abstract

The COVID-19 crisis is a global event that has created and amplified social inequalities, including an already existing and steadily increasing problem of employment and income insecurity and erosion of workplace rights, affecting workers globally. The aim of this exploratory study was to review employment-related determinants of health and health protection during the pandemic, or more specifically, to examine several links between non-standard employment, unemployment, economic, health, and safety outcomes during the COVID-19 pandemic in Sweden, Belgium, Spain, Canada, the United States, and Chile, based on an online survey conducted from November 2020 to June 2021. The study focused on both non-standard workers and unemployed workers and examined worker outcomes in the context of current type and duration of employment arrangements, as well as employment transitions triggered by the COVID-19 crisis. The results suggest that COVID-19-related changes in non-standard worker employment arrangements, or unemployment, are related to changes in work hours, income, and benefits, as well as the self-reported prevalence of suffering from severe to extreme anxiety or depression. The results also suggest a link between worker type, duration of employment arrangements, or unemployment, and the ability to cover regular expenses during the pandemic. Additionally, the findings indicate that the type and duration of employment arrangements are related to the provision of personal protective equipment or other COVID-19 protection measures. This study provides additional evidence that workers in non-standard employment and the unemployed have experienced numerous and complex adverse effects of the pandemic and require additional protection through tailored pandemic responses and recovery strategies.

## 1. Introduction

The COVID-19 crisis is a global event that has created and amplified social inequalities [1]. Regarding the labor market, for instance, unemployment soared, and work hours plunged, with ensuing substantial losses in labor income [2,3]. As a result, this pandemic caused an already existing, and steadily increasing, problem of employment and income insecurity and erosion of workplace rights to vastly deteriorate further, affecting workers across the world [4,5,6]. Of course, the degree of layoffs and corresponding loss of income was highly dependent on individual countries’ government containment measures, existing labor market regulations, ensuing labor market security, and worker rights.

This comes at a time when societal welfare systems, which have the potential to decommodify labor and buffer the pandemic’s impact on workers, families, and communities, are also being undermined due to years of the declining social safety net and a long-standing economic depression [7,8]. A stark consequence of weak welfare systems is the increasing economic inequality affecting societies globally, followed by an imbalance of power, and political influence that favors employers, especially in large profit-oriented corporations, to the detriment of the workers [9].

The generalized shock induced by the COVID-19 pandemic, including the deep and sudden economic recession that followed, led to complex public health, social, fiscal, and political crises, severely affecting labor [5], as briefly mentioned above, and increased the realization that employment could change dramatically even in environments previously perceived as relatively stable and predictable. Non-standard jobs were especially impacted by the crisis, given that the sectors most affected by COVID-19 containment measures tended to employ large shares of non-standard and informal workers [10,11]. Today, given the severe economic and labor market disruptions, non-standard forms of employment, such as gig work, are likely to continue to expand [3]. Non-standard workers (NSWs), generally identified as having part-time or flexible jobs, fixed temporary contracts, or being self-employed, as well as workers in the informal economy, lack the formal protections and benefits typically enjoyed by tenured workers with permanent, full-time positions [11,12,13]. Instead, non-standard employment (NSE) is frequently characterized by employment insecurity, income inadequacy, and a lack of rights and protection [13]. Although in times of stable economic conditions, flexible forms of employment are used to increase employment [14], in the context of the crisis, workers in this situation are likely to experience changes to their employment situation, a deterioration of work hours and income, unemployment, and poverty [2,15]. As Guy Ryder, the International Labour Organization (ILO)’s Director General, said in the early stages of the pandemic, “The crisis has uncovered the huge decent work deficits that still prevail in 2020 and shown how vulnerable millions of working people are when a crisis hits” [16].

This crisis also aggravated already existing health and well-being problems that are experienced by NSWs [17,18]. Additionally, NSWs in service sector occupations, health care, or agriculture, among others [17], are unable to work remotely, and thus, have a higher risk of infection—both on the way to work and at the workplace—than workers who can work remotely [19,20]. Their infection risk is further increased given that recurrent findings point to discrepancies in access to personal protection equipment (PPE), physical barriers, and information on safety measures against infection between non-standard and standard workers (SWs) [17]. On the other hand, the continuation of NSE, the dramatic life changes of those who lost their jobs, and subsequent risk of poverty following the lengthy and ongoing pandemic [20], are likely to add further stress and trigger diseases of despair, such as substance use disorders, mental health problems, and suicide attempts, among other consequences [21,22,23,24].

### 1.1. Country Context

Although facing the same virus, across the world, countries have responded to the COVID-19 pandemic with different mitigation policies and programs, both at the governmental and company level [11,25]. Such responses include a range of measures to *stimulate the economy and jobs, support enterprises, employment and incomes, protect workers in the workplace, and use social dialogue between government, workers and employers to find solutions* [26]. International studies that compare worker-related outcomes across countries allow us to get a glimpse into the diverse pandemic experiences of workers around the world and the level of protection they benefited from because of such country responses.

This study is focused on workers in six countries: Sweden, Belgium, Spain, Canada, the United States (U.S.), and Chile. All of them are capitalist economies that rely on private firms and competitive labor markets to organize economic activity. According to the *varieties of capitalism* approach to comparative political economy pioneered by Hall and Soskice, which was recently updated by Iverson and Soskice, the six countries are representative of three different models of economic organization, as shown in Table 1 [27,28]. Three of the countries (U.S., Canada, and Chile) are described as *Liberal non-coordinated market economies* (LME). Countries within this typology rely heavily on competitive markets to coordinate economic activity with minimal state regulation of labor markets. Firms rely on the labor market to supply general skills and have limited incentives to develop secure long-term employment relationships. Much employment tends to be “at will”, with both employers and employees relatively free to terminate relationships, and the prevalence of NSE tends to be relatively high. Two of the countries (Sweden and Belgium) are described as *Corporatist coordinated market economies* (CME). Countries within this typology rely less heavily on competitive markets to coordinate economic activity and rather more on non-market relationships, including information sharing through private networks and state supported initiatives. Employers rely more heavily on firm-specific skills generated by company training programs, and longer service-giving employers have more incentives to retain their workers in long-term relationships to protect their investment in skilled workers. Workers have more incentive to protect their employment to take advantage of their skills that are less valuable to other employers. Labor regulations tend to be supportive of more secure employment relationships. Employers are somewhat constrained from ending employment relationships without cause. The last country, Spain, is described as a *Non-coordinated market economy* (NME). The historical weakness of the Spanish welfare state limited its capacity to support the type of coordinated market economy found in Sweden and Belgium, whereas the predominance of neoliberal economic policy created weak labor market regulations and weak unions. The result is a heavy reliance on precarious employment arrangements and NSE.

Closely related to the economic typologies described above are welfare regime typologies [29], which can influence the nature of NSE by either buffering or reinforcing employment insecurities inherent in the different economic typologies. The six countries fall into five types of welfare regimes, as shown in Table 1. Two of the countries described as LME (U.S. and Canada) have *Anglo-Saxon Liberal Welfare Regimes* (LWR), with modest public social benefits and the provision of welfare through private markets and company benefit plans. This reinforces the commodification of labor increasing employment insecurity and income inequality. The other LME (Chile) has a welfare regime described as a *Latin American/Stratified Universalism Welfare Regime* (SUWR) that provides public social support only to certain segments of society, mainly workers in permanent full-time employment, and relies more heavily on the family to fill in gaps in the social wage. As above, this reinforces the commodification of NSE and increases the degree of their employment insecurity. The two countries described as CME have similar welfare regimes that tend to reduce labor commodification. Sweden is described as having a *Scandinavian Social Democratic Welfare Regime* (SDWR) where the state is the main provider of universal social benefits. This reinforces labor decommodification, reduces employment insecurity, and promotes a more equal distribution of income. Belgium’s *Continental/Conservative Welfare Regime* (CWR) also supports the decommodification of labor, but mainly for those in SE relationships. There is greater reliance on the family to provide social benefits and there are weaker income redistribution effects than in the case of Sweden. The final country, Spain, has been described as a *Mediterranean Post-Fascist Welfare Regime* (MWR) [30]. Social benefits are less fully developed and less equally distributed between core workers with good benefits and protections compared with those outside the core, including those in NSE.

At the start of the pandemic, the six countries were employing different models of political economy regarding state regulation of labor markets, approaches to social welfare policies, and the ways in which employers engage labor. These differences shape the occurrence of NSE, the level of employment insecurity of those in NSE, and the level of state support for labor. For instance, the combination of labor market regulations that shape employment security and characteristics of NSE across the six countries—such as strictness of employment protection of regular and temporary contracts, regulations of collective dismissals of regular contracts, rules for hiring temporary and fixed term contracts, and active or passive labor market expenditure—suggest that Belgium, Sweden, and to some degree, Spain, have the most secure employment and weaker labor commodification across the six countries, whereas Chile, the U.S., and Canada have the least secure employment and highest labor commodification [31,32]. Similarly, a mixture of labor market security and worker power indicators—such as prevalence of non-standard employment, bargaining strength (overall and for both regular and temporary employees), and public income support to working age populations—indicate that Sweden and Belgium have the most secure labor markets of the six countries and that their workers have the most bargaining power [31]. Moreover, the indicators for Chile, the U.S., and Canada are suggestive of higher labor market insecurity and weaker worker power, whereas Spain has the third highest labor regulations but low labor market security measures, which is suggestive of the underdeveloped role of the state in social support [31].

During the COVID-19 pandemic, these different political economies, with particular differences in welfare regimes, influenced how each of the six countries dealt with the crisis, with potentially different implications for NSWs and the unemployed, such as the extent of potentially negative health effects. Based on the combination of labor market regulations, security, and worker power indicators, we believe that for our country cases, workers in *corporatist coordinated market economies* and *social democratic and continental/conservative welfare regimes*, in countries such as Belgium and Sweden, would benefit from stronger protections during the pandemic, and thus, have better outcomes for all indicators. On the contrary, workers in *liberal non-coordinated market economies* and *Anglo-Saxon liberal and Latin American/stratified universalism welfare regimes*, such as the U.S., Canada, and Chile, would have access to weaker protections, and thus, experience the most hardship.

### 1.2. Study Purpose

The aim of this exploratory study, captured within the four research questions below, was to review employment-related determinants of health and of health protection during the pandemic. More specifically, this study aimed to examine several links between NSE and unemployment, and economic, health, and safety indicators during the COVID-19 pandemic in Sweden, Belgium, Spain, Canada, the U.S., and Chile, based on an online survey conducted by the Precarious Work Research (PWR) group from November 2020 to June 2021. PWR is an international group of researchers studying non-standard and precarious employment in the six countries mentioned above, through the use of various research methodologies including quantitative–qualitative mixed methods, systematic reviews, and participatory research approaches (precariousworkresearch.org, accessed on 5 May 2022). This collaboration is part of a 2019–2024 program funded by FORTE—the Swedish Research Council for Health, Working Life and Welfare, grant number 2019-01226.

The study focused on both NSWs and unemployed workers. Given the dynamic changes affecting workers’ employment during the COVID-19 crisis, in addition to focusing on workers’ current situations, based on the job held in the three months prior to survey completion, we also adopted a transition approach. Thus, two research questions focused on worker outcomes in the context of possible COVID-19-related employment transitions, and two considered the impact of current type and duration of employment arrangements.

- Are COVID-19-related changes to NSW employment arrangements, or unemployment, related to changes in work hours, income, and benefits?
- Are COVID-19-related changes to NSW employment arrangements, or unemployment, related to experiences of severe or extreme anxiety and depression?
- Are type and duration of employment arrangements, or unemployment, related to workers’ ability to cover regular expenses during the COVID-19 crisis?
- Are type and duration of employment arrangements related to the provision of PPE, information, training, or other COVID-19-related physical protection measures?

## 2. Materials and Methods

### 2.1. Study Sample

This study is based on six distinct convenience purposive samples of NSWs recruited from November 2020 to June 2021 from: Sweden (Stockholm County and Värmland County), Belgium (total area), Spain (Catalonia), Chile (three largest metropolitan areas: Santiago, Concepción, and Valparaíso), the U.S. (New York state) and Canada (Ontario province). The workers were recruited through different outreach methods, especially social media advertising (Supplementary Material S1). To qualify for inclusion, workers had to be 25–55 years old, based on the premise that typically, many workers in this age group have not recently entered, nor are they close to leaving, the labor market; however, it should be kept in mind that given the ongoing trends for increased education levels, with slight variations across countries, people are entering the labor market later, and thus, it is possible that some workers in our sample are at the beginning of their careers. Workers had to be either in NSE at the time of the survey, the three months preceding it, or unemployed, having lost their job permanently or temporarily due to COVID-19 pandemic related reasons. A worker was deemed to be in NSE if any of the following conditions were met: (i) not being employed directly, but rather being employed through a temporary help or staffing agency, self-employed without employees, and engaged in gig or platform work; or (ii) not working full-time; or (iii) not having an open-ended or permanent contract; or (iv) being in informal employment (defined as not paying taxes or without active pension contributions).

### 2.2. Data Collection

Data was collected through an online survey administered in the language(s) appropriate for each country’s population (See Supplementary Material S2 for an English version of the survey. Surveys in Spanish, Swedish, and Flemish are available upon request from the corresponding author). Except for one question from the EQ-5D-5L quality of life questionnaire [33] used to assess mental health, all other questions used in this study were designed for this survey, with the survey being pre-tested on a small scale in each country before launching. When necessary, questions were adjusted to fit the national/regional contexts. The country-specific modified question and answer categories are indicated along with the tables presenting those findings.

The surveys were launched at the beginning of November 2020 and stayed open until March 2021, except for Canada, where the survey was kept open until 30 June 2021, to allow for a larger sample. The minimum sample targeted for each country was 300 eligible participants. Each country obtained ethics approval: Sweden and Spain (Reg # 2020-02396, Karolinska Institute, Stockholm County, Sweden), Belgium (Ref# ECHW_228, Vrije Universiteit Brussel, Brussels, Belgium), Chile (Reg # 2020-012321, Pontificia Universidad Católica de Chile, Santiago, Chile), U.S. (REF# 2020-0412, Queens College, City University of New York, New York, NY, USA), and Canada (REB 20-110, MAP Centre for Urban Health Solutions, Li Ka Shing Knowledge Institute, Unity Health, Toronto, ON, Canada).

The survey information was collected using the RedCap (Sweden, Chile, Canada), Qualtrics (U.S., Belgium) and Alchemer (Spain) online survey software. Given existing risks linked to advertising surveys on social media sites, including the widespread filling of surveys by automatic bots or individuals filling the surveys multiple times to collect the incentive, we took several steps to ensure the quality of information collected. Thus, we only considered entries with valid phone numbers, postal codes, and email addresses and double-checked the similarity of answers entered within seconds or minutes from each other.

### 2.3. Study Variables

#### 2.3.1. Explanatory Variables

We used two employment variables to describe NSWs’ current situation, based on the job held in the three months prior to survey completion: employment arrangement and length or type of contract. In addition, we retrospectively identified five transitions to capture possible COVID-19 related changes (current situation vs. pre-COVID-19), based on several scenarios in which workers either maintained the same employment arrangement or transitioned between standard jobs, unemployment, and non-standard jobs. The five transitions are: (i) *same NSE arrangement*; (ii) *from unemployment to NSE*; (iii) *from one NSE to another NSE*; (iv) *from standard employment (SE) to NSE*; and (v) *became unemployed or furloughed due to COVID-19.* These transitions represent either preservation, improvement, or deterioration of employment arrangements and were identified based on participants’ answers to five survey questions. Four questions assessed workers’ SE or NSE arrangements within three months of survey completion and one assessed employment arrangements before the pandemic was declared (1 March 2020). The combination of employment type (SE, NSE, unemployed, or furloughed) and indication of job variation permitted the defining of the five transitions. The survey questions that facilitated the retrospective assignment of workers to employment transitions are listed in Supplementary Material S3.

#### 2.3.2. Outcome Variables

The outcome variables included employment, economic, and health, as well as occupational health and safety indicators. We used four employment indicators: (i) changes in work hours in the previous three months (Decreased a lot and Increased a lot); (ii) changes in work income in the previous three months (Decreased a lot and Increased a lot); (iii) new benefits or supports received (Yes, from employer and Yes, from government); (iv) previous benefits lost (Yes, from employer, Yes, from government, and Yes, other). We also used one economic indicator: having had difficulties covering regular expenses (such as food, rent, bills, etc.) during the previous three months (Yes, several times).

We used one item from the EQ-5D-5L quality of life questionnaire [33] to assess mental health (I am severely or extremely anxious or depressed); three indicators of occupational health and safety (OHS) protection for workers who worked close to potentially infected persons, assessing whether they were provided with (i) PPE such as masks and visors, (ii) information and training, and (iii) other measures such as physical barriers and social distancing. All three indicators had four categories which were dichotomized into Always vs. Sometimes, Never, and Not applicable.

Demographic characteristics were: (i) age (median age and three age groups: 25–31, 32–43, 44–55); (ii) gender (male, female, gender variant/non-binary, prefer not to answer); (iii) immigration status based on country of birth (immigrant, non-immigrant); and (iv) educational level (primary or less, completed secondary, and post-secondary).

Employment-related characteristics were: (i) type of employment arrangement (*employed directly by the employer, employed through a temporary help or staffing agency, self-employed without employees, and engaged in gig or platform work*); (ii) agreed employment or job contract length or type (*on-call or day-to-day basis, less than 6 months, 6 months to 1 year, longer than 1 year, permanent or open-ended, end date or length of job unknown)*; (iii) work hours (*part-time—i.e., less than 30 h per week, hours vary from week to week and could sometimes be less than 30, or full-time—i.e., 30 h or more per week*); and (iv) employment status (*formal, informal*).

Some of the outcome variables, such as changes in work hours, changes in work income, or suffering from anxiety or depression, had additional answer categories to those included in these analyses.

### 2.4. Data Analysis

We conducted a combination of descriptive and logistic regression analyses, including pooled logistic regressions. To maximize the sample sizes available for each analysis, participants with incomplete answers were not eliminated as long as they provided full answers to the questions related to current (within the three months prior to the completion of the survey) and pre-COVID-19 employment arrangements, which allowed us to identify employment transitions retrospectively. For this reason, sample sizes for each country differ by analysis, depending on the number of survey participants who provided answers to all questions involved in each respective analysis.

The variable capturing transitions in employment arrangements was used as stratification in the analysis of changes in work hours, work income, and benefits, given that these variables used the same timeframe, comparing the last three months before survey completion with before the start of the COVID-19 pandemic. In the case of economic difficulties, given that the question referred specifically to the three months preceding the survey, the use of current employment arrangements, rather than transitions, was most suitable for this analysis. Similarly, current employment arrangements were also used for the question about provision of PPE, information, training, or other COVID-19-related physical protection measures, since this information was specific to the pandemic. The question about depression or anxiety referred to the current situation at the time of the survey, however, given that typically, both these mental health issues are linked to changes in employment status, we used transitions in employment arrangements as stratification for this analysis.

We acknowledge that there are many factors impacting employment, including education level and language competencies, age, or gender, and we intended to examine the ways in which the employment conditions of worker sub-groups differ based on such unique identity factors; however, given the small numbers for each category, despite efforts to increase the diversity of our samples, we were not able to conduct any such disaggregated analyses.

Data analysis was conducted separately in each country, after agreeing on common inclusion and exclusion criteria, as well as coding and analysis protocols. The analysis was completed using Stata version 15.1 (Canada), Stata version 16 (Sweden, U.S.), SPSS v25 (Chile, Spain), and SPSS v28 (Belgium).

## 3. Results

### 3.1. Demographic and Employment Characteristics of the Convenience Sample

The demographic characteristics of the six country-specific convenience purposive samples are displayed in Table 2. The individual country samples ranged in size from 1444 in Spain to 1300 in Belgium, 1118 in Chile, 879 in Sweden, 447 in Canada, and 313 in the U.S., for a total of 5501 survey participants across the six countries. Overall, the country samples resembled each other with a few exceptions. The median age of the samples was between 33 and 40 years, with a higher proportion of females than males, and with most participants having post-secondary education across all six countries. The proportion of immigrants differed largely by country, from 10.7% in Chile to 41.5% in the U.S.

The employment characteristics, according to the job held in the three months prior to answering the survey, are shown in Table 3. Between 55% and 75% of all eligible survey participants were *employed directly; self-employment* was around 20% in Chile and the U.S.; and *gig/platform work* reached 10% in Canada and the U.S. Regarding the length or type of the contract, *on-call work* was most common in Sweden and the U.S., whereas in Belgium and Canada, most respondents had *permanent* jobs. In Spain and Chile, around 50% of the sampled individuals were in temporary jobs. Belgium had the highest percentage of *part-time* workers, followed by Spain and Canada, whereas in Chile, most respondents were *full-time* workers. *Informal* workers were very rare in European countries (<7%), whereas they were a majority in Chile (57%), they were around one third in the U.S., and one fifth in Canada. Overall, across all countries, when comparing the current employment situation with the pre-COVID-19 situation, most respondents had either maintained the *same NSE arrangement*, changed from *one NSE to another NSE*, or became *unemployed or furloughed* due to COVID-19. Two transitions showed precarization or worsening of workers’ employment arrangements, becoming *unemployed or furloughed* due to the pandemic, and moving *from SE to NSE.*

### 3.2. Employment and Economic Outcomes

#### 3.2.1. Changes in Work Hours and Work Income

**Figure 1:**
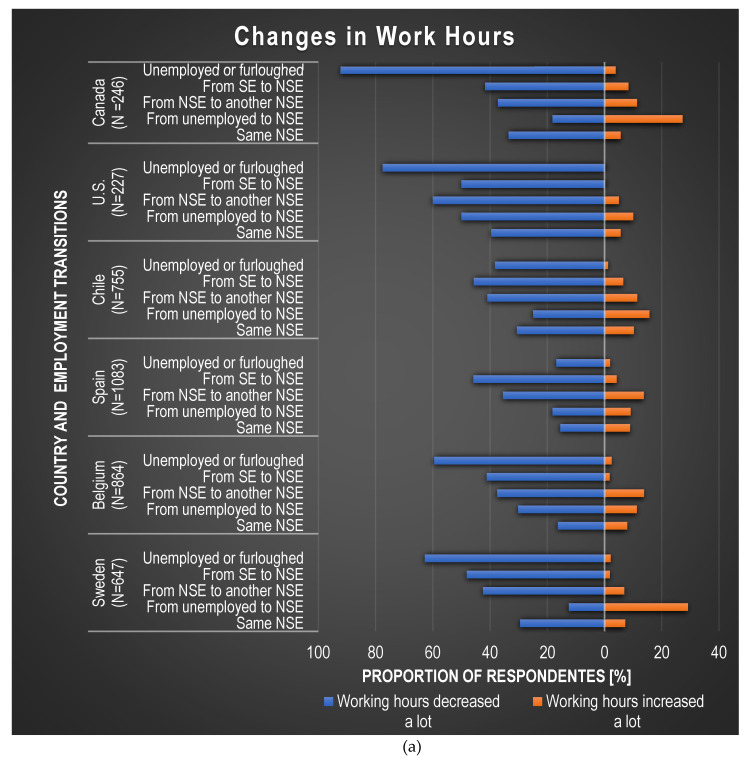

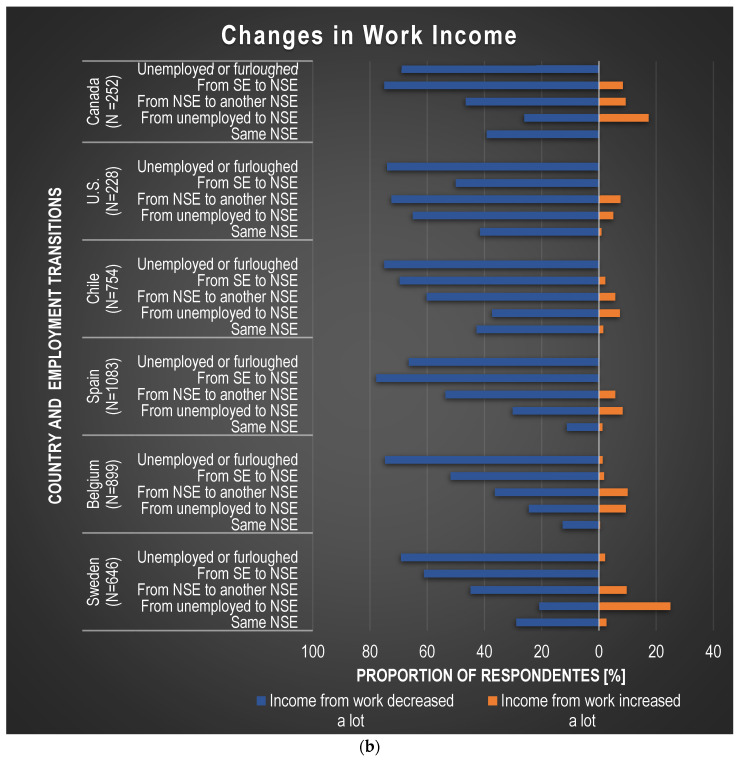

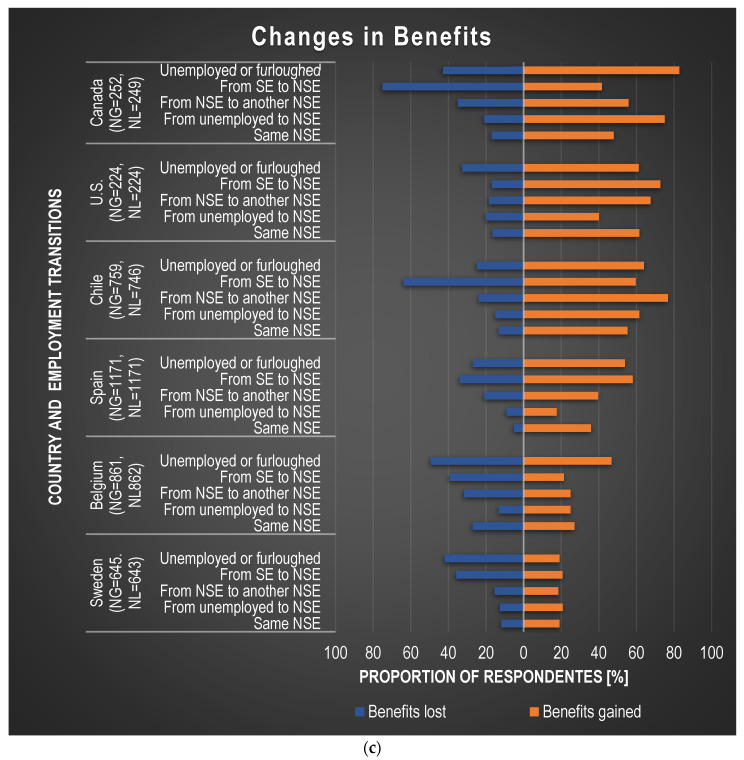
Proportion of respondents indicating changes in work hours, work income, and benefits compared to the job held or situation before the COVID-19 crisis, by country and by employment transition. (**a**) Changes in work hours, (**b**) Changes in work income, and (**c**) Changes in benefits. Notes: N represents the total sample of participants with jointly defined values for the employment transitions and changes in work hours, work income, and benefits questions. More specifically, for figure (**c**) NG represents the total sample of participants who gained benefits and NL the total sample of participants who lost benefits.

Figure 1 and Supplementary Material S4 show that, overall, 53% of workers experienced a reduction in work hours in the U.S., followed by Canada (39%), and Sweden (38%); over 56% of workers in the U.S. and Chile experienced a reduction in income, followed by Spain, Canada, and Sweden (40–44%). The smallest decreases in work hours across all five transitions were reported in Spain (21%) and Belgium (25%); and the smallest decrease in income was reported in Belgium (24%). The proportion of workers who gained work hours or income was less than 10% in all countries, being highest in the Americas. Work hours and income decreased in general for workers who went *from SE to NSE*, *from one NSE to another NSE*, or who *became unemployed or furloughed*. Workers who maintained *the same NSE* job were less likely to lose work hours and/or income, except for the U.S. where almost 40% of workers who kept the *same NSE* job indicated that their work hours decreased a lot.

#### 3.2.2. Changes in Benefits

A considerable proportion of workers in all countries received new benefits from employers and governments, which they were not entitled to before the pandemic, particularly so in Canada, the U.S., and Chile (56 to 62%), and to a lesser extent in Sweden, Belgium, and Spain (19% to 38%); however, at the same time, many workers reported losing previous benefits across countries. Those losing the most benefits were either *unemployed or furloughed*, or in a *SE to NSE* transition, with the most losses in this category being reported in Chile (64%) and Canada (75%).

#### 3.2.3. Difficulties Covering Regular Expenses

As shown in Table 4, the proportion of respondents who had difficulties covering regular expenses (such as food, rent, bills, etc.) at least several times during the three months preceding the survey ranged from 34% to 55%, with the highest proportion in American countries (50% or more) compared with European ones (35% to 40%). In general, individuals *becoming unemployed or furloughed due to COVID-19*, *gig or platform* workers, and workers *employed through temporary agencies* were those who most often had difficulties covering regular expenses (45% to 75%). The length or type of contract was associated with having difficulties covering regular expenses as well, with *on-call* workers being the worst off, especially in the U.S. and Chile.

### 3.3. Health and OHS Outcomes

#### 3.3.1. Workers Experiencing Anxiety or Depression

**Figure 2:**
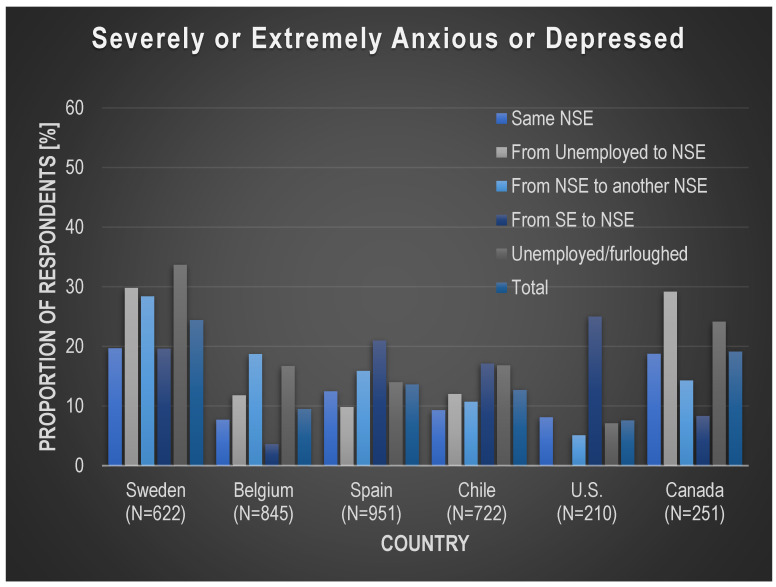
Proportion of respondents indicating that they are severely or extremely anxious or depressed, by country and by employment transition. Notes: For each country, N represents the total sample of participants with jointly defined values for the employment transitions and the self-declared anxiety and depression questions.

As shown in Figure 2 and Supplementary Materials S5, across all employment transitions, the percentage of workers who indicated that they were severely or extremely anxious or depressed was the highest in Sweden (24%) and Canada (19%), and the lowest in Belgium (10%) and the U.S. (8%). The employment transitions with the highest proportion of workers suffering from severe or extreme anxiety or depression differed by country.

**Figure 3:**
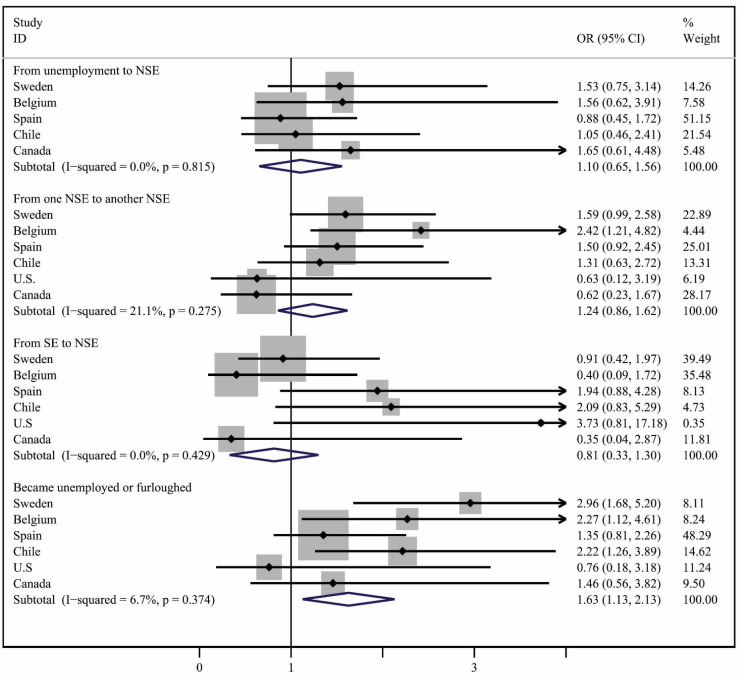
Pooled logistic regression analysis of predictors of severe or extreme anxiety or depression. Notes: SE = standard employment; NSE = non-standard employment arrangement. Reference category = same NSE. Model adjusted for age, gender, and education.

The unadjusted logistic regression models estimating the relationship between severe or extreme depression or anxiety and employment transitions show that, when compared with workers who had the *same NSE job* (indicating preservation of pre-COVID19 employment conditions)*,* the odds of reporting anxiety or depression were higher among the *unemployed or furloughed* in Sweden (OR = 2.07, CI 95% = 1.22–3.50), Belgium (OR = 2.38, CI 95% = 1.19–4.77), and Chile (OR = 1.96, CI 95% = 1.14–3.38), and among workers moving from *one NSE to another NSE* in Sweden (OR = 1.61, CI 95% = 1.01–2.57) and Belgium (OR = 2.73, CI 95% = 1.41–5.28) (Supplementary Material S6). No clear patterns of higher odds of reporting anxiety/depression according to employment transitions were found in Spain, the U.S., or Canada. These odds changed only slightly after adjusting for age, gender, and educational attainment (Supplementary Material S6 and Figure 3). Thus, when compared with workers who maintained the *same NSE arrangement*, the odds of reporting severe or extreme anxiety or depression increased slightly for workers who became *unemployed or furloughed* in Sweden (OR = 2.96, CI 95% = 1.68–5.21) and Chile (OR = 2.22, CI 95% = 1.26–3.89), and decreased slightly in Belgium (OR = 2.27, CI 95% = 1.12–4.61). The odds decreased slightly for workers moving from *one NSE to another NSE* both in Sweden (OR = 1.59, CI 95% = 0.99–2.58) and Belgium (OR = 2.42, CI 95% = 1.21–4.83).

#### 3.3.2. Access to OHS Protective Measures against COVID-19

As shown in Table 5*,* across all employment arrangements, close to half of the respondents who indicated that they worked in close proximity to infected persons reported that their employer had consistently provided them with PPE, the percentage ranging from 39% to 49% in four of the six countries, whereas in Canada, the numbers were higher (62%), and in Sweden, they were much lower (18%). Slightly lower percentages of workers were found in Belgium, Chile, the U.S., and Canada, and slightly higher percentages of workers were found in Sweden and Spain, who reported that they were provided with appropriate information and training to prevent them from becoming infected. Even lower percentages of workers in Belgium, Spain, Chile, and Canada, and slightly higher percentages of workers in Sweden and the U.S., were provided with other protective measures against COVID-19, such as physical barriers and social distancing.

When comparing the provision of these protective measures across employment arrangements, Belgium, Chile, the U.S., and Canada showed higher proportions among workers who were *employed directly*, whereas in Sweden and Spain, they were among those *employed through a temporary agency*. Given that we did not know the employment arrangements of workers in the *unemployed or furloughed* category, we did not include them in this analysis.

## 4. Discussion

### 4.1. Main Findings

The results of our analysis suggest that, in our samples, COVID-19-related changes in NSW employment arrangements, or unemployment, are related to changes in work hours, income, and benefits, as well as the self-reported prevalence of suffering from severe to extreme anxiety or depression. More specifically, work hours and income decreased in general for workers in three transitions: *from SE to NSE*, *from one NSE to another NSE*, or those who *become unemployed or furloughed,* and were less likely to change for workers who maintained the *same NSE*, except for the U.S. Most benefits were lost by workers in a *SE to NSE* transition or who *became unemployed or furloughed* due to the pandemic. Regarding workers experiencing anxiety or depression, when compared with workers who had the *same NSE job,* the self-reported prevalence of suffering from severe or extreme anxiety or depression increased for all other transitions, with a few exceptions.

Furthermore, our results suggest a link between worker type and duration of employment arrangement, or unemployment, and ability to cover regular expenses during the pandemic. In our samples, workers who had difficulties covering regular expenses were most often those who became *unemployed or furloughed due to COVID-19*, who were *gig or platform* workers, and workers who were *employed through temporary agencies.* The length or type of the contract was also associated with having difficulties covering regular expenses, with *on-call* workers being worse off. In addition, our findings indicate that the type and duration of employment arrangements are related to the provision of personal protective equipment, information, training, or other COVID-19 physical protection measures. For instance, in four of the six countries, a higher proportion of workers *employed directly* had access to such resources, whereas in two of them, Sweden and Spain, they were mostly available to workers *employed through a temporary agency*.

Despite such similarities in findings across the six countries included in this study, there are also several key differences. Such differences could be linked to the distinct economic conditions, labor markets, and political contexts characterizing each country. For instance, the levels of informal employment differed significantly between European (2–7%) and American continent countries (30–57%), suggesting an Atlantic divide. Such a divide appears to correspond with the distinct economic organization models and welfare regime typologies and is probably the indicator that best accounts for the true differences between labor markets across the six countries included in this analysis. It seems that that the economic and welfare typologies discussed earlier partially shaped outcomes. Workers in countries employing *liberal non-coordinated market economies* and/or *Anglo-Saxon liberal welfare regimes* generally reported the highest frequency of job loss, work hour loss, income loss, and difficulties covering expenses, whereas workers in countries employing *corporatist coordinated market economies* and *social democratic* or *continental/conservative welfare regimes* generally had fewer negative outcomes. Overall, these findings seem to support our initial expectations about the links between economic and welfare regime typologies and worker outcomes in our country cases; however, some of the findings also suggest that other factors, not captured by economic and welfare typologies, such as short-term responses to COVID-19, may have played a role in outcomes such as depression and anxiety, where both Canada, a *liberal non-coordinated market economy*, and Sweden, a *corporatist coordinated market economy*, had the highest percentages of severely or extremely anxious or depressed participants. Another possibility is that in Sweden, the high prevalence of depression or anxiety among respondents was due to the realization that even in an environment previously perceived as relatively stable and predictable, their employment situation could change considerably.

### 4.2. Interpretation

The widespread adoption across countries of full and partial lockdown, and social distancing measures that were meant to slow the spread of COVID-19 infections to minimize the loss of human lives and shield national health systems from collapsing under the weight of the pandemic, caused significant disruption and solvency challenges to both small and large businesses [2]. Of course, the impact on businesses differed based on their size, sector, and capacity to adapt to online work and platform-based services [34]. In turn, the disruption caused to businesses impacted workers in numerous ways [2,34] and the findings of this study show a glimpse into these effects on non-standard workers in six high-income countries.

Renewed attention placed on the worsening employment conditions and labor market problems, especially in light of the COVID-19 pandemic [35], could accelerate positive transformation, facilitating more equal and resilient societal relations, which, in turn, could be of crucial value from the wider perspective of achieving a sustainable society [5,20,36].

#### 4.2.1. Employment and Economic Outcomes

According to previous studies, the impact of the pandemic on work hours is ambiguous. On one hand, the pandemic has increased the share of employees working from home, which has been related to longer working hours, including working in the evenings and on weekends [37]. On the other hand, the pandemic led to a decrease in work hours, as a result of both temporary or permanent cutbacks on work time, worker dismissals, and displacement. This is as a result of employment contraction affecting labor markets during the pandemic, especially in certain sectors of the economy (e.g., service sector) that have been hit the hardest [2,38].

The findings in this survey indicate that work hours, across all countries, decreased in general for NSWs, especially for those who experienced precarization or worsening of employment arrangements, and including those who transitioned from *SE* *to NSE* or who became *unemployed or furloughed* due to COVID-19. Interestingly, workers who moved *from unemployment to NSE* also declared a decrease in work hours. This exception could be explained by a mismatch between the reference points and the timing of the unemployment. More specifically, workers may have experienced a pandemic related reduction in work hours, followed by both the loss of that job and the move to another job. These findings support ILO’s estimates for 2020 that 8.8% of global work hours would be lost relative to the fourth quarter of 2019 [2]. Although in March 2022, during the finalization of this manuscript, and approximately two years after the start of the COVID-19 pandemic, there are signs of recovery in certain labor markets and sectors, the overall global recovery is sluggish and uncertain, given cyclical outbreaks followed by setbacks [3]. ILO’s 2021 recovery projections have since deteriorated, with newer estimates indicating that overall, work hours and labor force participation are expected to stay at pre-pandemic levels at least until 2023 [3]. This situation is especially worrisome for workers exposed to employment insecurity and those who do not benefit from rights and protections in relation to employment, such as NSWs.

The reductions in economic activities and work hours are undeniably followed by a loss of income for workers and a worsening of poverty levels, which puts workers affected by income insecurity, especially those with no income replacement, at a higher risk of poverty compared with workers in SE [2]. In our study, across all countries, we found the highest losses in income among employees who experienced a worsening of their employment arrangements, such as those who *became unemployed or furloughed* and those who moved from a *NSE to another NSE*. These findings are supported by a joint ILO OECD report, which mentioned that many of the workers who did not lose their jobs lost work hours and were affected by wage cuts, particularly those with less secure work arrangements and those concentrated in hard-hit industries [39]. Interestingly, despite common wage decreases, the report revealed that several countries, such as the U.S. and Canada, experienced an overall increase in average wages due to the greater loss of employment among low-paid workers, which, in turn, raised the average wages of those who kept their jobs [39].

The pandemic has also made evident the gaps in social protection coverage across countries since, globally, only a third of workers have access to sickness benefits and a fifth of those who lose their job have unemployment benefits [20]. In our study, for instance, it was mostly the workers who became *unemployed or furloughed*, and in Canada and Chile, those who switched *from one NSE to another NSE* job who lost the most benefits, possibly because they had the weakest social contracts. The typical lack of benefits affecting workers in NSE was also reflected in our survey, given that a high percentage of workers in Chile, the U.S., and Canada indicated that they gained new benefits during the pandemic. Not surprisingly, in the absence of regular benefits available to NSWs, these countries had to mobilize the most ad-hoc benefits. The findings related to gaining new benefits showed a different pattern for European countries, making evident the European/American continent labor market typology division.

Given workers’ loss of work hours, income, and benefits, it is not surprising that, across all countries, between one third and half of our respondents faced difficulties covering basic living expenses such as food, rent, and bills during the three months preceding the survey. Another unsurprising finding from our survey was that *temporary agency* workers and those with shorter contracts and *on-call* work, who typically experience high levels of employment and income insecurity, had the most difficulties making ends meet. Without adequate social and economic support being adopted as part of the COVID-19 economic response and recovery efforts, these workers find themselves in difficult positions, especially in those countries with weaker welfare systems that lack sufficient universal socio-economic protections. This situation is further worsened by extremely competitive housing markets, with low affordability levels and high rents, characterizing most of the analyzed countries [40,41,42,43].

#### 4.2.2. Health and OHS Outcomes

Our findings suggest that it is not only workers who lost their jobs, but also those who changed jobs or moved from unemployment to employment, that had a higher risk of experiencing severe or extreme anxiety or depression. Moreover, there is the additional possibility that feelings of stress and anxiety during the pandemic increased in the general population, since they are a response to the perceived threat of the pandemic, including the fear of becoming infected [44]. Additionally, such symptoms could be the result of a pandemic prompted exacerbation of known determinants of poor mental health [44], including the existence of co-morbidities, access to food, and living conditions. For instance, a 2020 study using data from the UK Household Longitudinal Study found that mental health deterioration, compared with pre-pandemic trends, was unequal among population sub-groups, with a higher increase in levels of mental distress experienced by people living with young children and those living in low-income homes [45]. Moreover, anxiety or depression could be triggered by any new situation and change in lifestyle [46], or by employment status or job change, irrespective of job quality [47]; however, these findings can also mean that the NSE arrangements that the workers in our samples moved into may have been of poorer quality, were more insecure than the preceding ones, or were carrying a larger risk of exposure to, and infection by, COVID-19. Another explanation is that feelings of insecurity may continue to be present in individuals who already lost their job once, suggesting that the time gone by has not been able to remediate psychological discomfort from past unemployment. In addition, in some cases, unemployed individuals may have a more stable source of income or access to better basic benefits than workers with some forms of NSE [11]. Somewhat related, although losing one’s job or becoming furloughed could both be stressful, given that financial protection and employment security could be quite different in each situation, by combining the unemployed or furloughed categories in our analyses, we may have missed a much higher prevalence of mental health difficulties among the unemployed. Although the conduct of sensitivity analyses to differentiate between the two categories of workers could have revealed valuable insights, given that the number of workers who were unemployed and furloughed in the U.S. and Canada, the two countries with smallest samples, were quite small—52 and 54, respectively—and the number of people suffering from severe or extreme anxiety or depression in this transition were 3 and 7, respectively, we refrained from breaking these categories down even further, and instead, analyzed them together.

Given that the employment transitions with the highest percentage of workers reporting symptoms of anxiety or depression are different across countries, we conclude that it is not just the transition itself that affects outcomes but the country specific context and the nature of non-standard work within that context. For instance, given that temporary work is less common in several of the six countries (e.g., Belgium and Sweden), whereas in others (e.g., Spain) it is more common, and unemployment during the pandemic had a more temporary nature in countries such as Belgium, changes in employment status during the COVID-19 crisis may have resulted in different mental health effects for workers in the six countries.

We reviewed the pre-pandemic baseline levels of anxiety and depression in the six countries of interest to better understand if the high rates we found in Sweden and Canada are perhaps explained by high pre-pandemic levels; however, the only common source of data we found for all six countries is a WHO database that provides 2015 population-based estimates for the prevalence of depression [48]. According to this database, the highest rates of depression were in the U.S. (5.86) and Spain (5.21), followed by Chile (5.01) and Sweden (4.88), with the lowest rates found in Belgium (4.81) and Canada (4.65), whereas in our data, the highest rates are in Sweden (24.4) and Canada (19.1), followed by Spain (13.6) and Chile (12.7), and the lowest rates are observed in Belgium (9.5) and the US (7.6). Moreover, according to a Eurostat database, in 2019, Sweden had the second highest rate among European countries (11.7), higher than both Belgium (7.3) and Spain (5.7) [49]. Furthermore, based on the OECD, the pre-pandemic depression rates were 10.8 in Sweden, 9.5 in Belgium, and 6.6 in the U.S., whereas anxiety rates were 14.7 in Sweden, 11 in Belgium, and 8.2 in the U.S [50]. Unfortunately, OECD data for Canada and Spain is missing for both anxiety and depression rates [50]. The inconsistencies within these findings, and the lack of comparable country rates for pre- and post-pandemic levels, is not surprising given that: (i) survey instruments used to assess depression may differ between countries, and sometimes from year to year [50]; (ii) the individual country samples we use in our study are not representative of each respective country’s overall population; and (iii) in our analysis, we focused only on severe or extreme anxiety and depression.

The availability of support and worker protection measures are also very important. For instance, in addition to having higher risks of losing their jobs and experiencing decreases in work hours and income, workers in NSE typically lack basic protections such as social and health benefit coverage that workers in SE usually have [20]. These forms of exclusion have several significant implications. First, these workers may experience barriers when accessing social and health services [51,52], especially when such services are not universal and when they are accompanied by service charges [20]. Second, during the pandemic, in addition to challenges related to access to vaccination, early diagnosis, and treatment services, such barriers can pose difficulties in terms of access to mental health services [20,53]. This situation was further worsened by the fact that the demand for such services increased during the pandemic for some at-risk groups who had a higher probability of experiencing mental health issues [54,55]. Third, the lack of paid sick days commonly experienced by workers in NSE, although with differences noted across the six countries, could further contribute to the exacerbation of stress and mental health symptoms since workers realize that if or when they get sick, they have no income replacement.

In addition, as identified in a recent literature review, the percentage of workers in NSE that work in economic sectors or occupations with a high risk of COVID-19 infections, including service industries such as retail and food preparation and serving, agriculture, transport, construction, health, and domestic services, is higher than in other sector and occupations [17]. This situation increases NSE workers’ risk of contracting the virus, especially if they are not consistently provided with PPE and other health-protective measures. The broad variation in workers’ access to protective measures across the six countries studied, and across the five employment transitions, further confirms the importance of the country-specific context and, in this case, the overall adoption of public health measures at a country level. Although we were not able to find sources of comparative data across the six countries included in this analysis with regard to the provision of personal protective equipment, we referred to information included in the WHO’s review of country policy responses and the COVID-19 Stringency Index collated by a team at the University of Oxford [56,57]. According to these two data sources, Belgium and Sweden adopted the least restrictive policies to limit the spread of COVID-19 [26,57]. Additionally, deficiencies regarding the provision of PPE equipment, including in sectors with a high risk of transmission, such as health or long-term care [58,59], are also documented in the literature. Overall, among the six countries in this analysis, Belgium and Sweden had some of the lowest scores, and Canada and Chile had some of the highest scores on the COVID-19 Stringency Index (0 to 100, 100 = strictest), which is a composite measure consisting of nine indicators, including the closing of schools, restrictions on public gatherings, travel bans, testing policies, and contact tracing. For instance, on January 31 2021, about midway through the data collection period, the scores for the six countries were as follows: Belgium (62.96), Sweden (69.44), Spain (71.3.), U.S. (71.76), Canada (75.46), and Chile (79.17) [57].

Thus, the low percentage of workers who had access to PPE or other protective measures in countries such as Sweden, does not necessarily reflect the workers’ employment arrangements or the sample of respondents, but the broader public health approach when responding to the pandemic. Although we do not know the specific reason, we believe that the unexpected finding in Catalonia, Spain, that a higher percentage of workers employed through temporary agencies were provided with COVID-19 protective measures than those hired directly, may be due to the fact that temporary agency workers are often hired in economic sectors, such as the service sector [60], which are more likely to be in-person jobs that did not stop their activities, and thus, those workers may have been provided with access to such measures before everyone else. Moreover, in Sweden something slightly similar is observed; however, this result must be taken with caution given that the sample sizes and differences are small. Finally, the fact that we do not find the same results in other countries may be explained by differences in the characteristics of the country samples, or specific regulations put in place in Spain, that merit further study.

### 4.3. Strengths and Weaknesses

This study allowed us to get a glimpse into the impact of the COVID-19 pandemic on NSWs across multiple countries. The collection of information through these similar and simultaneous surveys allowed us to hear directly from relatively high numbers of unemployed workers and NSWs with different levels of employment precarity. Given the unprecedented nature of the COVID-19 crisis, the possibility of gathering relatively timely information about its various effects on NSWs is a definite advantage since such information can be used to not only inform short-term government and employer mitigation responses, but also long-term recovery strategies. Furthermore, knowing the importance of assessing the evolution of the labor market with accuracy, surveys such as this one, which obtain this information directly from workers, are a valuable tool, since they complement existing estimating models that predict, for instance, cumulative hours worked, along with other indicators of economic activity [2].

Although there are concerns regarding the reliability of online surveys, it appears that web-based questionnaires can have equal or higher reliability than traditional application modes [61], including measures of mental health [62]. Furthermore, being self-administered instruments, online surveys have been described to make it easier for subjects to provide answers to sensitive subjects [62], which is an important benefit given the sensitive nature of some of the survey questions, including those about the formal or informal character of workers’ employment, income, potential financial difficulties, mental health concerns, and health protection measures provided by their employers; however, a limitation of online surveys is related to higher non-response rates [61], compounded by our use of social media advertising to promote the surveys, which requires reading comprehension skills, internet access, literacy, and active use of social media sites, which could vary by country, education level, and age group. Such requirements may have inadvertently eliminated voices of relevant eligible participants with unique experiences and different types of outcomes from our study. Similarly, although in some countries, the surveys were translated in more than one language, relevant participants who did not speak the languages in which the surveys were administered may have been missed. Although this limits the representativity of our data, we do not anticipate there to have been differences in the association between exposure and outcome in respondents, compared with non-respondents, and hence, we do not anticipate having introduced bias into our analyses.

Although we did not aim for very large and representative samples, we acknowledge that a key limitation of our study comes from the use of convenience purposive samples for each country, which are non-representative of the whole population, partly due to lower sample size and self-selection bias. Furthermore, due to challenges with recruiting eligible participants, the sample sizes for Canada and the U.S. were considerably smaller than those of other countries. In addition, despite our ongoing efforts and targeted strategies to increase the number of eligible respondents for some demographics (e.g., gender variant or non-binary, men, informal workers, and lower-educated workers) and levels of employment precarity categories, these numbers remained relatively small. This limitation prevented us from conducting disaggregated analyses to understand the ways in which each sub-group of workers was differently affected by the pandemic, based on their unique social identity and employment precarity factors. Furthermore, small numbers in all categories, other than those employed directly, make it difficult to give meaning to some percentages and may explain some of the inconsistent findings across countries. When planning our samples, we intentionally only focused on unemployed/furloughed or non-standard workers, in an effort to learn more about this worker sub-group, which is often less studied than the standard worker group; however, an implication of this is that we cannot compare findings among non-standard and standard workers.

Moreover, the accuracy of our findings may be affected by self-reporting and recall bias, and given that the survey was open for longer in one of the six countries, the effects of the pandemic on workers may appear more profound than in countries that had earlier data collection. Similarly, given the cyclical nature of the COVID-19 crisis and the distinct characteristics of its various phases, some of the worker outcomes specific to the data collection phase may have been different if collected during other pandemic phases. Additionally, although we acknowledge a range of country-specific context factors that could have an impact on the economic, health, and well-being outcomes experienced by the workers in our samples, we do not control for any of these factors, including the welfare regimes or economic models, in our analyses. Furthermore, since we do not have baseline data for our samples, we are not able to compare the COVID-19 findings with pre-pandemic findings. Similarly, given the purposive convenience nature of our cross-sectional samples, we do not use country-level baseline data as a comparison point either, except for a brief review of pre-pandemic levels of depression and anxiety within the six countries involved in this analysis. Furthermore, comparing the intensity and pattern of transitions before and after the pandemic could have provided valuable information to the understanding of employment insecurity experienced by non-standard workers. Unfortunately, given that the survey data collected allows the study of workers’ job transitions as only being triggered by the pandemic, but not their job transitions before the pandemic, we are not able to estimate if COVID-19 has changed the intensity and the pattern of transitions.

Additionally, we acknowledge that several of our analyses are descriptive, except for the logistic regression analyses, the establishment of employment transitions, and their use as a stratification method. Despite this, given that the pandemic is ongoing and there are currently many unknown aspects about its impact on non-standard workers, we believe that our manuscript makes an important contribution to this topic, and the results of the descriptive analyses, the use of transitions, and the logistic regression analyses contribute valuable insights, thus, expanding existing research on this topic. The information contained in the descriptive tables, covering changes in work hours and work income, changes in benefits, difficulties covering regular expenses, provision of COVID-19 protection measures specific to NSWs and the unemployed, is unique because to the best of our knowledge, it has not been collected among similar populations or among multiple countries anywhere else and it comes directly from workers, thus, complementing information obtained through estimation models.

## 5. Conclusions

Overall, the findings from this study provide additional evidence that workers in non-standard employment experience numerous and complex adverse effects of the pandemic and need additional protection. The results suggest that countries should adopt policies that enable workers to retain their job during a crisis, as this study revealed that changing either from standard to non-standard employment, or between two non-standard employment arrangements during the COVID-19 crisis, is related to negative effects on employment, economic, and health outcomes. In addition, although many countries have provided temporary economic, health, and social benefits to workers in non-standard employment to mitigate the effects of the pandemic and soften its blow, the longer term or permanent adoption of such benefits after the crisis would contribute to improved equity across categories of workers and reduce labor market inequities.

When planning the pandemic response and recovery strategies, these should be tailored to ensure they respond to the unique needs of worker sub-groups, especially those groups with many insecurities such as non-standard workers, who typically have minimal social protection benefits and are at high risk of poverty. In addition to providing worker protection, such measures would stimulate and enable an inclusive economic and social recovery that could boost labor demand. As of today, the opposite has been done in many instances, and thus, the most vulnerable workers have been the least likely to benefit from the pandemic responses.

## Figures and Tables

**Table 1:** Division of countries according to variety of capitalism or model of economic organization and welfare regime typology.

Country	Variety of Capitalism	Welfare Regime Typology
Sweden	Corporatist coordinated market economies	Social democratic
Belgium		Continental/conservative
Spain	Non-coordinated market economy	Mediterranean post-fascist
Canada	Liberal non-coordinated market economies	Anglo-Saxon liberal
United States		
Chile		Latin American/stratified universalism

**Table 2:** Demographic characteristics of the convenience purposive samples across the six countries.

Characteristics	Sweden	Belgium	Spain	Chile	U.S.	Canada
**Convenience Survey Sample**	**N = 879**	**N = 1300**	**N = 1553**	**N = 1118**	**N = 313**	**N = 447**
**Age**	**N = 879**	**N = 1300**	**N = 1553**	**N = 1118**	**N = 313**	**N = 447**
Median Age (IQR)	36 (29–47)	38 (31–48)	33 (28–42)	40 (31–48)	40 (33–47)	40 (31–48)
25–31	34.9%	25.8%	45%	25.1%	18.2%	26.9%
32–43	31.5%	39.4%	32.6%	35.5%	45.4%	35.6%
44–55	33.6%	34.%	22.3%	39.4%	36.4%	37.6%
**Gender**	**N = 879**	**N = 1300**	**N = 1553**	**N = 1118**	**N = 313**	**N = 447**
Male	22.6%	23.5%	33.3%	32.9%	31%	19.9%
Female	68.9%	74.6%	64.3%	63.8%	62%	72.9%
Gender variant/non-binary	1.4%	0.5%	1.4%	0.5%	0.9%	2.7%
Prefer not to answer	7.1%	1.3%	1%	2.8%	6.1%	4.4%
Immigration Status *	**N = 819**	**N = 1290**	**N = 1543**	**N = 1092**	**N = 311**	**N = 433**
Immigrant	19.9%	15.5%	18%	10.7%	41.5%	15%
Non-immigrant	80.1%	84.5%	82%	89.3%	58.5%	85%
Education Level *	**N = 819**	**N = 1290**	**N = 1541**	**N = 1087**	**N = 307**	**N = 429**
Primary	5.1%	20.%	16.2%	2.7%	9.8%	1.4%
Secondary	21.4%	36.8%	23.4%	19.7%	22.5%	11.9%
Post-secondary	73.5%	41.8%	60.5%	77.6%	67.7%	86.7%

Notes: IQR: Interquartile range (quartile 25–quartile 75). N represents the total sample of participants who provided answers to a respective question. * The following variables have missing values: immigration status and education level.

**Table 3:** Employment characteristics and employment transitions of the convenience purposive samples across the six countries.

Characteristics	Sweden	Belgium	Spain	Chile	U.S.	Canada
Employment Arrangement *	**N = 703**	**N = 1182**	**N = 1266**	**N = 736**	**N = 246**	**N = 384**
Employed directly by the employer	70.6%	74.1%	76.2%	59.9%	55.3%	72.7%
Employed through a temp agency	9.7%	17.9%	16.1%	15.4%	13.8%	7%
Self-employed with no employees	12.7%	6.3%	7.2%	22.1%	20.3%	10.2%
Gig/platform work	7.1%	1.6%	0.5%	2.6%	10.6%	10.2%
Agreed Contract Length or Type *	**N = 703**	**N = 1182**	**N = 1266**	**N = 736**	**N = 246**	**N = 384**
On-call or day-to-day basis	34.9%	4.7%	11.7%	13.2%	30.1%	15.9%
Less than 6 months	20.9%	14.2%	24.6%	29.9%	14.6%	21.4%
6 months to 1 year	18.1%	11.2%	23.9%	22.7%	6.9%	8.1%
Longer than 1 year	5.6%	8.0%	12.7%	0 **	9%	11.2%
Permanent or open-ended	12.9%	52.2%	13.3%	17.4%	23.9%	32.3%
End date or length of job unknown	2.3%	1.1%	9.6%	3.8%	9%	5.2%
Not applicable	5.4%	7.3%	4.1%	13%	6.5%	6%
Work Hours *	**N = 703**	**N = 1182**	**N = 1266**	**N = 736**	**N = 246**	**N = 384**
Part-time (<30 h per week)	37.8%	53.6%	39.1%	24.5%	37.8%	39.1%
Hours vary from week to week (could sometimes be <30)	25.2%	17.1%	10.6%	24.5%	32.5%	23.4%
Full time (≥30 h per week)	36.98%	29.4%	50.3 %	51.1 %	29.7%	37.5%
Formal/Informal *	**N = 703**	**N = 1182**	**N = 1266**	**N = 736**	**246**	**384**
Formal	98.4%	97.1%	93.5%	43.1%	68.7%	80%
Informal	1.6%	2.9%	6.5%	56.9%	31.3%	20.1%
Employment Transitions *	**N = 647**	**N = 886**	**N = 1171**	**N = 764**	**232**	**296**
Same NSE	47.1%	68.7%	44.3%	34.9%	46.1%	51.7%
From unemployment to NSE	7.4%	6.1%	12.6%	14.5%	8.6%	9.8%
From one NSE to another NSE	22.6%	9.5%	20.1%	11.8%	17.7%	16.2%
From SE to NSE	8.4%	6.7%	4.3%	6.2%	5.2%	4.1%
Became unemployed or furloughed due to COVID-19	14.5%	9%	18.7%	32.6%	22.4%	18.2%

Notes: SE = standard employment; NSE = non-standard employment. N represents the total sample of participants who provided answers to a respective question. * All variables in this table (employment arrangement, agreed contract length or type, work hours, formal/informal, and employment transitions) have missing values. Employment arrangement, agreed contract length or type, work hours, and formal or informal status are based on the job held in the three months prior to survey completion. The employment transitions were identified retrospectively and capture possible COVID-19 related changes based on several scenarios in which workers either maintained the same employment arrangement or transitioned between standard jobs, unemployment, and non-standard jobs. ** For the Chilean survey, for the question assessing the agreed contract length, the category ‘Longer than 1 year’ was eliminated because in the Chilean context it is the same as ‘Permanent or open-ended’.

**Table 4:** Proportion of respondents who had difficulties covering regular expenses at least several times during the three months prior to survey completion, by employment arrangement, length or type of contract, and by country.

Difficulties Covering Regular Expenses													
	Country	Sweden N = 645		Belgium N = 886		Spain N = 979		Chile N = 840		U.S. N = 213		Canada N = 253	
		n	%	n	%	n	%	n	%	n	%	n	%
Employment arrangement (including unemployed or furloughed)	Total	228	35.4	301	34.0	400	40.9	460	54.8	117	54.9	127	50.2
	Employed directly	115	28.8	177	30.4	206	36.1	145	42.9	46	51.1	71	43.8
	Employed through a temp agency	25	47.1	67	45.6	52	38.2	49 *	58.3	14	58.3	12	66.7
	Self-employed with no employees	21	32.8	14	27.5	26	41.9	55	45.5	20	51.2	10	43.5
	Gig or platform work	22	61.1	3	18.8	1	33.3	12	75	11	64.7	14	66.7
	Unemployed or furloughed due to COVID-19	45	48.4	40	44.9	115	55.6	199	70.8	26	60.5	20	69
Contract length or type (including unemployed or furloughed)	Total	228	35.4	301	34.0	400	40.9	460	54.8	116	54.7	127	50.2
	On-call or day-to-day basis	87	44.6	20	50.0	50	52.1	53	67.9	40	71.4	15	46.9
	Less than 6 months	29	25.4	60	44.1	85	41.7	85	51.8	14	53.8	26	53.1
	6 months to 1 year	22	22.2	23	24.5	50	25.1	41	31.5	5	45.5	13	54.2
	Longer than 1 year	10	32.2	21	32.8	14	31.8	0	0	3	23.1	10	45.5
	Permanent or open-ended	22	28.6	122	30.0	43	37.7	41	40.6	19	45.2	31	43.7
	End date or length of job unknown	5	45.4	1	16.7	28	33.3	9	47.4	7	70	6	46.2
	Not applicable	8	33.3	14	28.0	15	48.4	32	47.8	3	25	6	46.2
	Unemployed or furloughed due to COVID-19	45	48.4	40	44.9	115	55.6	199	70.8	26	60.5	20	69

Notes: For the Canadian survey, the question about difficulties covering regular expenses was slightly different, asking if respondents had difficulties paying for housing during the previous 6 months; participants included this analysis are those who answered ‘Yes, all the time’ and ‘Yes, some of the time’. The total N sample for each country consists of participants with jointly defined values for the employment arrangement and contract length, respectively, and for the difficulties covering the regular expenses questions. The n sample represents the number of survey participants who indicated that they had difficulties covering regular expenses. Both n and the % are shown by employment arrangement, contract length or type, and by country, as well as a total per employment arrangement and per contract length or type. * In Chile, this category corresponds to subcontract workers (not temporary agency workers).

**Table 5:** Proportion of respondents who worked near infected persons indicating whether they consistently received PPE, information, training, or other protection measures to prevent them from becoming infected, by country and by employment arrangement.

	Provision of PPE, Information, Training, or Other Physical Protection Measures										
Characteristics	Country	Employed Directly		Employed through a Temp Agency		Self-Employed with No Employees		Gig or Platform Work		Total	
	N	n	%	n	%	n	%	n	%	n	%
Provided with personal protective equipment (e.g., masks, visors, etc.)	Sweden (N = 294)	40	17.5	7	21.2	1	6.3	4	25.0	52	17.7
	Belgium (N = 415)	168	50.8	27	40.9	6	46.2	2	40.0	203	48.9
	Spain (N = 391)	118	37.8	28	47.5	4	21.1	0	0	150	38.4
	Chile (N = 183)	58	53.2	15 *	42.9	10	34.5	0	0	83	45.4
	U.S. (N = 62)	19	52.8	3	37.5	4	36.4	2	28.6	28	45.2
	Canada (N = 87)	47	65.3	4	50	2	66.7	1	25	54	62.1
Provided with appropriate information and/or training	Sweden (N = 294)	40	17.5	8	24.2	3	18.8	5	31.3	56	19.1
	Belgium (N = 416)	149	44.9	18	27.3	5	38.5	2	40.0	174	41.8
	Spain (N = 391)	130	41.7	29	49.2	4	21.1	0	0	163	41.7
	Chile (N = 183)	55	50	9 *	26.5	10	34.5	1	10	75	41
	U.S. (N = 62)	13	36.1	2	25	3	27.3	3	42.9	21	33.9
	Canada (N = 87)	37	51.4	2	25	2	66.7	1	25	42	48.3
Provided with other measures (e.g., physical barriers, social distancing, etc.)	Sweden (N = 294)	47	20.5	5	15.2	3	18.8	3	18.8	58	19.7
	Belgium (N = 416)	139	41.9	20	30.3	4	30.8	2	40.0	165	39.7
	Spain (N = 391)	102	32.7	21	35.6	6	31.6	0	0	129	33.0
	Chile (N = 182)	45	41.3	7 *	20.6	7	24.1	1	10	60	33
	U.S. (N = 62)	15	41.7	1	12.5	5	45.4	2	28.6	23	37.1
	Canada (N = 87)	32	44.4	2	25	1	33.3	1	25	36	41.4

Notes: The total N sample for each country represents the participants who specified that they worked in close proximity to infected persons and who had jointly defined values for the questions about employment arrangements. Each of the three questions were related to personal protective equipment, information, and physical protection measures. The n sample represents the number of survey participants who indicated that they were provided with personal protective equipment, information, and physical protection measures. Both n and the % are shown by country and by employment arrangement, as well as a total per country across all employment arrangements. PPE = personal protection equipment. * In Chile, this category corresponds to subcontract workers (not temporary agency workers).

## Data Availability

De-identified participant data from the PWR study cannot be made publicly available for ethical and legal reasons. It could, however, be made available to researchers who meet the criteria for access to confidential data, after approval from all ethics committees, providing approval for this research is obtained. If interested, contact the corresponding author: virginia.gunn@utoronto.ca.

## Supplementary Materials

The following supporting information can be downloaded at: https://www.mdpi.com/article/10.3390/ijerph19105865/s1, Supplementary Material S1: Outreach methods used across the six countries to recruit participants for the survey; Supplementary Material S2: English Version Survey; Supplementary Material S3: Retrospective employment transition identification; Supplementary Material S4: Respondents indicating changes in work hours, income, and benefits when compared with the job held or situation before the COVID-19 crisis, by country and by employment transition; Supplementary Material S5: Proportion of respondents indicating they are severely or extremely anxious or depressed, by country and by employment transition; Supplementary Material S6: Logistic regression models of predictors of severe or extreme anxiety or depression, by country and by employment transition.
