# Supplementary material for: Non-Standard Employment and Unemployment during the COVID-19 Crisis: Economic and Health Findings from a Six-Country Survey Study
Source: Int J Environ Res Public Health. 2022 May 11;19(10):5865. doi: 10.3390/ijerph19105865 (PMC9140645; doi:10.3390/ijerph19105865)
Supplement: Supplementary file 1 [file ijerph-19-05865-s001.zip › ijerph-1656701-supplementary.pdf]

Supplementary Material

Supplementary Material S1: Outreach methods used across the six countries to recruit participants for the survey

Country	Summary of outreach methods
**Sweden**	Paid adds via Facebook (FB), Instagram (IG) and Alkompis* network website. Survey promotion by e-mail/phone directly to groups on FB, unions, and associations with common interests in NSE.
**Belgium**	FB groups and paid ads and survey promotion through a (politically left-wing) union representing commerce, finance, industry, and services sectors.
**Spain**	Social media, but mainly through FB ads.
**Chile**	FB and IG paid ads, survey promotion through university site and social networks: FB, IG, Twitter (TW).
**U.S.**	Social media (paid ads on FB, university TW accounts); announcement on day laborer radio program; and emails to community-based organizations, worker/worker health organizations, occupational health and public health listservs, and personal contacts.
**Canada**	Paid adds via FB, IG, and Google Ads, Employment News, KIJIJI**. Survey promotion to organizations and individuals through messaging on TW and other social media and by email/phone to organizations with common interests in NSE.

Notes: NSE = non-standard employment. * Arabic speaking community platform. ** Canadian online classified advertising website.

Supplementary Material S2: English Version Survey

A few initial questions

1. **What is your age? ____________** (If not between 25-55 **end survey**)
2. **Which State/Region/district do you live in?** (If relevant and/or exclusion criteria)
3. **Have you worked for pay or profit at any time in the past 3 months?**
    1. Yes, and currently working (Go to Q6)
    2. Yes, but currently not working (Go to Q6)
    3. No (Go to Q4)
4. **What was the reason for not working?**
    1. Unemployed (Go to Q5)
    2. Furloughed (Go to Q5)
    3. On temporary leave (e.g., Sick leave, parental leave) (**End survey**)
    4. Other (**End survey**)
5. **Did you lose your work permanently or temporarily after the COVID19 outbreak (1st of March)?**
    1. Yes (Go to Q7-9, then skip to Q18 contact information and continue to part 2)
    2. No (**End survey**)
6. **Which of the following best describes the job/contract that paid you the most in the last 3 months)?**
    - The employment relation in that job
        1. Employed directly by the employer from whom I work
        2. Employed through a temporary help/staffing agency
        3. Self-employed with no employees
        4. Self-employed with employees
        5. Gig/platform work
    - The agreed length of that job (from when the current agreement or contract started until when it will end)
        1. On-call or day-to-day basis
        2. Less than 6 months
        3. 6 months to 1 year
        4. Longer than 1 year
        5. Permanent/Open-ended
        6. Don't know
        7. Not applicable
    - The working hours per week for that job
        1. Part-time, less than 30 hours per week
        2. Hours vary from week to week and could sometimes be less than 30
        3. Full-time, 30 hours or more per week
    - That job pays taxes, makes pension contributions
        1. Yes
        2. No

[If employed directly by the employer AND permanent/open-ended contract AND full-time AND Yes to paying taxes & making pension contributions = **End survey**]

Background questions

7. **I am… / How do you describe yourself?**
    1. Man
    2. Woman
    3. Gender variant / non-conforming
    4. Prefer not to answer
8. **Were you born in country of site?** [Yes/No]
    - **(Optional) Were both your parents born in country of site?** [Yes/No]
    - **(Optional) Year of immigration**
    - **(Optional) Do you identify as a non-white racialized worker?** [Yes/No]
9. **What is the highest grade or year of school you have completed?**
    1. Did not complete primary school
    2. Primary school graduate (9-10)
    3. High school graduate (2-3)
    4. Some College / University education
    5. College / University graduate

The following questions apply to the job that paid you the most <u>in the last 3 months</u>

10. **[Only if Q3=1 OR Q3=2] What was your income (after taxes and deductions) from the work you did <u>in the past month or the last month you worked</u>? If you don't know the exact amount, please provide your best guess.** [<30%<60% of median/60-79%/80-119%/120-159%/160-199%/>200%]

Belgium	Euro	28773/year
Canada	Canadian Dollar	47831/year
Chile	Chilean Peso	4936008/year
Catalonia	Euro	20141/year
Sweden	Swedish Krona	315465/year
United States	US Dollar	38330/year

11. **[Only if Q3=1 OR Q3=2] In the past 3 months, how much did your work income vary from month to month?**
    1. Very much
    2. Somewhat
    3. A little
    4. Not at all
12. **[Only if Q3=1 OR Q3=2] In the past 3 months, how often did you work on an on-call basis? (That is, you had no set schedule, and you were contacted only when there was work)**
    1. Always
    2. Often
    3. Sometimes
    4. Rarely
    5. Never
    6. Not applicable

13. **[Only if Q3=1 OR Q3=2] How likely will your total working hours be reduced in the next 3 months?**
    1. Very likely
    2. Likely
    3. Somewhat likely
    4. Not likely
    5. Not likely at all
    6. Don't know
    7. Not applicable
14. **[Only if Q3=1 OR Q3=2] Do/did you know your work schedule at least one day in advance?**
    1. Always
    2. Often
    3. Sometimes
    4. Rarely
    5. Never
    6. Not applicable
15. **[Only if Q3=1 OR Q3=2] Do/did you get paid if you miss/ed a day's work because of illness, attending family affairs, personal reasons…?**
    1. Always
    2. Often
    3. Sometimes
    4. Rarely
    5. Never
    6. Not applicable
16. **[Only if Q3=1 OR Q3=2] Do/did you feel afraid to demand better employment and working conditions at work (e.g., pay, benefits, schedule, safety measures, etc.)?**
    1. Always
    2. Often
    3. Sometimes
    4. Rarely
    5. Never
    6. Not applicable
17. **[Only if Q3=1 OR Q3=2] Indicate how often you can/could take the following actions without obstacles or consequences**
    - Take sick leave
    - Go to the doctor
    - Take vacations
    - Request a day off for family affairs
    - Request a day off for personal reasons

    1. Always
    2. Often
    3. Sometimes
    4. Rarely
    5. Never
    6. Not applicable

18. **If you would like to be considered for an interview and/or follow-up survey, please provide the following so we can contact you** [MULTIPLE CHOICE: Yes to survey/Yes to interview/No]

Your name: ________________
Phone number: ________________
Email: __________________
Facebook messenger or other means of communication you expect to be using for the foreseeable future: ______________________

Do you know anyone who might be interested in being part of this study? If you do, could you forward the survey link to them or share it on social media Survey link: PWR Screening survey

Part 2
Work and the Covid-19 outbreak

The next part of the survey is about the impact on Covid-19 and your work and health
[Next/continue] (NO OPT OUT – ONLY DROP OUT)

The next questions are about the impact of the COVID19 outbreak. Be aware that the lockdown measures in country of site started on X March 2020. If we say "before the outbreak" we mean the period before X March.

19. **[Only if Q3=1 or Q3=2] Which of the following best describes the job/contract that paid you the most before the COVID19 outbreak in country of site?**
    1. It was the same job as the one I've had in the past 3 months and the arrangement was the same (Go to Q21)
    2. It was the same job as the one I've had in the past 3 months, but the arrangement was different
    3. It was another job than the one I've had in the past 3 months
    4. I was unemployed (Go to Q21)
20. **[Only if Q3=3 OR Q19=2 OR Q19=3] Please describe the job/contract that paid you the most before the COVID19 outbreak in country of site.**
    - The employment relation in that job
        1. Employed directly by the employer from whom I work
        2. Employed through a temporary help/staffing agency
        3. Self-employed with no employees
        4. Self-employed with employees
        5. Gig/platform work
    - The agreed length of that job (from when the agreement or contract started until its end)
        1. On-call or day-to-day basis
        2. Less than 6 months
        3. 6 months to 1 year
        4. Longer than 1 year

5. Permanent/Open-ended
6. Don’t know
7. Not applicable

- The working hours per week for that job
  1. Part-time, less than 30 hours per week
  2. Hours vary from week to week and could sometimes be less than 30
  3. Full-time, 30 hours or more per week
- That job pays taxes, makes pension contributions
  1. Yes
  2. No

21. **[Hide if Q4=unemployed OR Q4=furloughed] Have you been unemployed/furloughed at any point <u>since the COVID19 outbreak</u>?** [MULTIPLE CHOICE: Yes, unemployed/Yes, furloughed/No]
22. **During the past 3 months, have you been working more or less hours per week than you normally did <u>before the COVID19 outbreak</u>?**
    1. Much decreased
    2. Somewhat decreased
    3. More or less unchanged
    4. Somewhat increased
    5. Much increased
    6. Not applicable
23. **During the past 3 months, has your income from work changed compared to <u>before the COVID19 outbreak</u>?**
    1. Much decreased
    2. Somewhat decreased
    3. More or less unchanged
    4. Somewhat increased
    5. Much increased
    6. Not applicable
24. **Have you received benefits or assistance during the COVID19 outbreak that you, under non-pandemic circumstances, would not be eligible to receive (local examples)?** [MULTIPLE CHOICE: Yes, from employer/Yes, from government/Yes, other/No]
    - **[If any “Yes” option] Please specify**
25. **Have you lost benefits, assistance, or entitlements during the COVID19 outbreak compared to before the outbreak (local examples)?** [MULTIPLE CHOICE: Yes, from employer/Yes, from government/Yes, other/No]
    - **[If any “Yes” option] Please specify**
26. **During this pandemic, have you received all the benefits you were entitled to?** [Yes/Yes but with delay/No/I don’t know]
    - **[If No] Please specify why not**
27. **Were you isolated/quarantined at home or elsewhere during the COVID19 outbreak because you had COVID19 or were exposed to someone with COVID19?** [Yes/No]
    - **[If Yes] Did you receive income, sick or family leave benefits for this time period?**
    1. Yes, I received my full usual income
    2. Yes, I received part of my usual income

3. No

28. **During the pandemic, did you work in close proximity to COVID19-infected persons or met many persons/crowds (such as in healthcare, transport, retail, etc.)** [Yes/No/Not applicable]
    - **[If Q28=Yes] Were you provided with personal protection equipment (such as masks, visors, etc.)?** [Always/Sometimes/Never/Not applicable]
    - **[If Q28=Yes] Were you provided with appropriate information and/or training to protect you from being infected?** [Always/Sometimes/Never/Not applicable]
    - **[If Q28=Yes] Were other measures taken to protect you from being infected (such as barriers, social distancing, etc.)?** [Always/Sometimes/Never/Not applicable]
29. **By what means did you usually commute to work during the outbreak?** [MULTIPLE CHOICE: Walk/Bike/Car/Car-sharing/Public transport/Motorbike or scooter/Worked from home/Did not work/Other]

Household and economic situation

30. **How well does this statement describe your current employment situation: "I have chosen my employment arrangement because it suits the needs of myself and/or my family"?**
    1. Strongly disagree
    2. Disagree
    3. Neither agree nor disagree
    4. Agree
    5. Strongly agree
31. **Who do you live with?** (MULTIPLE CHOICE)
    1. I live alone
    2. My partner or spouse
    3. My child/children
    4. My parent(s)
    5. My extended or blended family (siblings, others)
    6. Friend(s)/flat mate(s)
    7. Other
32. **How many persons including yourself and children are living in your household?** [Number]
33. **Hoy many rooms does the household have, excluding the kitchen and bathrooms?** [Number]
34. **What was your total household income from all sources (wages or salary, welfare, unemployment benefits, social security, child support, etc.) after taxes and deductions (net) <u>in the past month</u>? If you don't know the exact amount, please provide your best guess.** [<30%<60% of median/60-79%/80-119%/120-159%/160-199%/>200%]

Belgium	US Dollar/year	35 142
Canada	US Dollar/year	34 831
Chile	US Dollar/year	16 489
Spain/Catalonia	Euro/year	33 321
Sweden	US Dollar/year	33 834.8
United States	US Dollar/year	50 291.7

35. **During the past 3 months, have you had difficulties in covering your regular expenses (such as food, rent, bills, etc.)?** [Yes, several times/ Yes, once/No]

36. **Would you or your household be able to pay an unexpected expense of half median salary of country within a month without borrowing or asking for help?** [Yes/No]

Divide the value in the table by 24 and round to closest 100EUR/USD-ish. For Belgium the number would be 1200 EUR, Sweden 13000 SEK, etc.

Belgium	Euro	28773/year
Canada	Canadian Dollar	47831/year
Chile	Chilean Peso	4.936.008/year
Spain	Euro	17121/year
Sweden	Swedish Krona	315465/year
United States	US Dollar	38330/year

Health and well-being

37. **Do you smoke cigarettes?**
    1. Yes, one or more cigarettes a day
    2. Yes, occasionally (less than one cigarette a day)
    3. No, I quit smoking
    4. No, I have never smoked
38. **Have you ever been diagnosed with any of the following health conditions?** (MULTIPLE CHOICE)
    1. Hypertension
    2. Diabetes
    3. Chronic obstructive pulmonary disease (COPD)
    4. Asthma
    5. Depression
    6. Anxiety disorder
    7. None of the above
39. **What is your height in cm/in?** [Number (local measure)]
40. **What is your weight in kg/pound?** [Number (local measure)]

EQ5D-5L. Under each heading, please tick the ONE box that best describes your health TODAY.

41. **Mobility**
    1. I have no problems in walking about
    2. I have slight problems in walking about
    3. I have moderate problems in walking about
    4. I have severe problems in walking about
    5. I am unable to walk about
42. **Self-care**
    1. I have no problems washing or dressing myself
    2. I have slight problems washing or dressing myself
    3. I have moderate problems washing or dressing myself
    4. I have severe problems washing or dressing myself
    5. I am unable to wash or dress myself
43. **Usual activities (e.g., work, study, housework, family, or leisure activities)**
    1. I have no problems doing my usual activities
    2. I have slight problems doing my usual activities

3. I have moderate problems doing my usual activities
4. I have severe problems doing my usual activities
5. I am unable to do my usual activities

44. **Pain/discomfort**
    1. I have no pain or discomfort
    2. I have slight pain or discomfort
    3. I have moderate pain or discomfort
    4. I have severe pain or discomfort
    5. I have extreme pain or discomfort
45. **Anxiety/depression**
    1. I am not anxious or depressed
    2. I am slightly anxious or depressed
    3. I am moderately anxious or depressed
    4. I am severely anxious or depressed
    5. I am extremely anxious or depressed

46. **We would like to know how good or bad your health is TODAY. This scale is numbered from 0 to 100. 100 means the best health you can imagine. 0 means the worst health you can imagine. Mark an X on the scale to indicate how your health is TODAY.**

    Now, please write the number you marked on the scale in the box below.

Thank you for completing the survey!

Is there anything else you would like to tell us about how the COVID19 outbreak affected your working life and how you managed it? [Free text box]

Do you know anyone who might be interested in being part of this study? If you do, could you forward the survey link to them or share it on social media Survey link: PWR Screening survey

[Only if End survey]
Thank you (Not matching inclusion criteria)

You do not match the inclusion criteria, thank you for participation.

Supplementary Material S3: Retrospective employment transition identification

**Pre-COVID-19**	**Current**<br>(Within the 3 months prior to survey completion)	**Transition Type**	**Survey questions and answers used to retrospectively identify workers' employment transitions**	
NSE	NSE	Same NSE	Which of the following best describes the job/contract that paid you the most before the COVID-19 outbreak?	It was the same job as the one I've had in the past 3 months and the employment arrangement was the same.
NSE	NSE	Same NSE		It was the same job as the one I've had in the past 3 months, but the employment arrangement was different. *
Unemployment	NSE	From unemployment to NSE	Which of the following best describes the job/contract that paid you the most before the COVID-19 outbreak?	I was unemployed.
NSE	NSE	From one NSE to another NSE	Which of the following best describes the job/contract that paid you the most before the COVID-19 outbreak?	It was another job than the one I've had in the past 3 months*.
SE	NSE	From SE to NSE	Which of the following best describes the job/contract that paid you the most before the COVID-19 outbreak?	It was the same job as the one I've had in the past 3 months, but the employment arrangement was different. *<br>or<br>It was another job than the one I've had in the past 3 months. *
SE<br>or<br>NSE	Unemployed or furloughed	Became unemployed or furloughed due to COVID	Reason for not working for pay or profit at any time in the past 3 months	Unemployed<br>or<br>Furloughed

Notes: NSE - Non-standard employment arrangement, defined as (i) not being employed directly by an employer, (ii) not working full-time, (iii) not having an open-ended or permanent contract, or (iv) being in informal employment (defined as not paying taxes or not making pension contributions). SE - Standard employment, defined as having a permanent or open-ended contract, full-time hours, and having paid taxes and pension contributions. The five transitions capture five possible scenarios in which workers either maintained the same employment arrangement or moved between standard jobs, unemployment, and non-standard jobs, representing either preservation, improvement, or deterioration of employment conditions. Four questions assessed workers' standard or non-standard employment arrangements within three months of survey completion (Q3, Q4, Q5, Q6 with 4 subsections), and one assessed the employment arrangements pre-COVID-19 (before the pandemic was declared on March 1, 2020) (Q 20 with 4 subsections). The questions are listed in Supplementary Material S4 - English Version Survey. *Workers

selecting categories that indicated a different employment arrangement pre-COVID-19 pandemic were asked another question to further assess that arrangement (Q 20 with 4 subsections).

Supplementary Material S4: Respondents indicating changes in work hours, income, and benefits when compared to the job held or situation before the COVID-19 crisis, by country and by employment transition

Changes in Work Hours, Income, and Benefits													
Characteristics	Country	Same NSE		From unemployment to NSE		From one NSE to another NSE		From SE to NSE		Became unemployed or furloughed due to COVID-19		Total	
	N	n	%	n	%	n	%	n	%	n	%	n	%
Work hours decreased a lot	Sweden (N=647)	90	29.5	6	12.5	62	42.5	26	48.2	59	62.8	243	37.6
	Belgium (N=864)	97	16.3	16	30.2	30	37.5	23	41.1	47	59.5	213	24.7
	Spain (N=1083)	75	15.5	24	18.2	75	35.5	22	45.8	35	16.9	231	21.3
	Chile (N=755)	81	30.6	27	25	36	40.9	21	45.7	95	38.3	260	34.4
	U.S. (N=227)	42	39.6	10	50	24	60	6	50	38	77.5	120	52.8
	Canada (N =246)	48	33.6	4	18.2	16	37.2	5	41.7	24	92.3	97	39.4
Work hours increased a lot	Sweden (N=647)	22	7.21	14	29.2	10	6.9	81	1.9	2	2.1	49	7.6
	Belgium (N=864)	47	7.9	6	11.3	11	13.8	1	1.8	2	2.5	67	7.8
	Spain (N=1083)	43	8.9	12	9.1	29	13.7	2	4.2	4	1.9	90	8.3
	Chile (N=755)	27	10.2	17	15.7	10	11.	3	6.5	3	1.2	60	7.9
	U.S. (N=227)	6	5.6	2	10	2	5	0	0	0	0	10	4.4
	Canada (N=246)	8	5.6	6	27.3	5	11.6	1	8.3	1	3.9	21	9.3
Income from work decreased a lot	Sweden (N=646)	88	28.9	10	20.8	65	44.8	33	61.1	65	69.2	261	40.4
	Belgium (N=865)	76	12.7	13	24.5	29	36.3	29	51.8	59	74.7	206	23.8
	Spain (N=1083)	122	11.2	40	30.1	113	53.6	36	77.8	148	66.4	449	41.5
	Chile (N=754)	112	42.7	41	37.3	53	60.2	32	69.6	186	75	424	56.2
	U.S. (N=228)	44	41.5	13	65	29	72.	6	50	37	74	129	56.6
	Canada (N=250)	56	39.2	6	26.1	20	46.5	9	75	20	69	111	44.4
Income from work increased a lot	Sweden (N=646)	8	2.6	12	25	14	9.7	0	0	2	2.1	36	5.6
	Belgium (N=865)	2	0.3	5	9.4	8	10.0	1	1.8	1	1.3	17	2.0
	Spain (N=1083)	6	1.2	11	8.3	12	5.7	0	0	0	0	29	2.7
	Chile (N=754)	4	1.5	8	7.3	5	5.7	1	2.2	0	0	18	2.4

	U.S. (N=228)	1	0.9	1	5	3	7.	0	0	0	0	5	2.2
	Canada (N=250)	0	0	4	17.4	4	9.	1	8.3	0	0	9	3.6
Gained new benefits (from employer and government)	Sweden (N=645)	58	19.1	10	20.8	27	18.5	11	20.8	18	19.1	124	19.2
	Belgium (N=862)	161	27.1	13	25.0	20	25.0	12	21.4	37	46.8	243	28.2
	Spain (N=1171)	186	35.8	26	17.6	93	39.6	29	58.0	118	53.9	452	38.6
	Chile (N=759)	147	55.3	67	61.5	69	76.7	28	59.6	158	64	469	61.8
	U.S. (N=224)	64	61.5	8	40	27	67.5	8	72.7	30	61.2	137	61.2
	Canada (N=252)	69	47.9	18	75	24	55.8	5	41.7	24	82.8	140	55.6
Lost previous benefits (from employer, government, and other sources)	Sweden (N=643)	36	11.8	6	12.5	22	15.3	19	35.8	39	41.9	122	19
	Belgium (N=861)	161	27.1	7	13.2	25	31.6	22	39.3	39	49.4	254	29.5
	Spain (N=1171)	65	12.5	22	14.9	49	20.9	17	34.0	59	26.9	212	18.1
	Chile (N=746)	34	13.1	16	15	21	23.9	30	63.8	84	24.8	185	24.8
	U.S. (N=224)	17	16.5	4	20	7	18.4	2	16.7	16	32.6	46	20.5
	Canada (N=249)	24	16.9	5	20.8	15	34.88	9	75	12	42.9	65	25.9

Notes: SE = standard employment; NSE = non-standard employment. The total N sample for each country consists of participants with jointly defined values for the employment transitions indicator and each respective outcome indicator. The n sample represents the number of survey participants who indicated changes in work hours, income from work, and gained or loss benefits, by country, by employment transition, and as a total per country for all employment transitions. Red font is used to represent the %, which is calculated by country, by employment transition, and as a total per country across all employment transitions.

Supplementary Material S5: Proportion of respondents indicating they are severely or extremely anxious or depressed, by country and by employment transition

Severe or Extreme Anxiety or Depression												
Country	Same NSE		From unemployed to NSE		One NSE to another NSE		From SE to NSE		Becoming unemployed or furloughed due to COVID-19		Total	
N	n	%	n	%	n	%	n	%	n	%	n	%
Sweden (N=622)	58	19.7	14	29.8	40	28.4	10	19.6	30	33.7	152	24.4
Belgium (N=812)	43	7.7	6	11.8	14	18.7	2	3.6	12	16.7	77	9.5
Spain (N=1023)	50	10.9	12	9.7	31	15.6	9	19.6	27	13.8	129	13.6
Chile (N=722)	24	9.3	13	12	9	10.7	7	17.1	39	16.8	92	12.7
U.S. (N=210)	8	8.1	0	0	2	5.1	3	25	3	7.1	16	7.6
Canada (N=251)	27	18.8	7	29.2	6	14.3	1	8.3	7	24.1	48	19.1

Notes: SE = Standard employment; NSE = non-standard employment. The total N sample for each country consists of participants with jointly defined values for the employment transitions indicator and anxiety or depression indicator. The n sample represents the number of survey participants who indicated that they suffered by severe or extreme anxiety or depression, by country, by employment transition, and as a total per country for all employment transitions. Red font is used to represent the %, which is calculated by country, by employment transition, and as a total per country across all employment transitions.

Supplementary Material S6: Logistic regression models of predictors of severe or extreme anxiety or depression, by country and by employment transition

Model 1 - Employment Transition (Unadjusted)

		Severe or Extreme Anxiety or Depression			
Country	**Explanatory Variables**	**Odds Ratio**	**P Value**	**95% CI Inf**	**95% CI Sup**
**Sweden**	From unemployment to NSE	1.726	0.120	0.867	3.434
**N = 622**	From one NSE to another NSE	1.611	0.044	1.011	2.566
	From SE to NSE	0.992	0.984	0.469	2.097
	Became unemployed or furloughed due to COVID-19	**2.068**	0.007	1.223	3.497
**Belgium**	From unemployment to NSE	1.588	0.318	0.641	3.932
**N = 809**	From one NSE to another NSE	**2.733**	0.003	1.414	5.282
	From SE to NSE	0.441	0.267	0.104	1.871
	Became unemployed or Furloughed due to COVID-19	**2.381**	0.014	1.190	4.765
**Spain**	From unemployment to NSE	0.876	0.697	0.451	1.702
**N = 1023**	From one NSE to another NSE	1.509	0.095	0.931	2.446
	From SE to NSE	1.990	0.086	0.907	4.364
	Became unemployed or Furloughed due to COVID-19	1.315	0.285	0.796	2.170
**Chile**	From unemployment to NSE	1.165	0.711	0.519	2.616
**N = 722**	From one NSE to another NSE	1.329	0.437	0.649	2.718
	From SE to NSE	1.999	0.138	0.800	4.993
	Became unemployed or Furloughed due to COVID-19	**1.962**	0.015	1.140	3.377
**U.S.**	From unemployment to NSE	-			
**N = 192**	From one NSE to another NSE	0.614	0.550	0.124	3.033
	From SE to NSE	3.791	0.080	0.851	16.878
	Became unemployed or Furloughed due to COVID-19	0.875	0.849	0.220	3.474
**Canada**	From unemployment to NSE	1.784	0.244	0.673	4.729

**N = 251**	From one NSE to another NSE	0.722	0.507	0.276	1.887
	From SE to NSE	0.394	0.382	0.049	3.183
	Became unemployed or Furloughed due to COVID-19	1.379	0.507	0.534	3.558
**Model 2 - Employment Transition (Adjusted for Age, Gender, Education)**					
Country	**Explanatory Variables**	**Odds Ratio**	**P Value**	**95% CI Inf**	**95% CI Sup**
**Sweden N = 621**	From unemployment to NSE	1.530	0.246	0.745	3.143
	From one NSE to another NSE	1.594	0.057	0.986	2.578
	From SE to NSE	0.912	0.816	0.423	1.967
	Became unemployed or Furloughed due to COVID-19	**2.956**	<0.001	1.679	5.205
**Belgium N = 809**	From unemployment to NSE	1.561	0.342	0.623	3.912
	From one NSE to another NSE	**2.417**	0.012	1.211	4.825
	From SE to NSE	0.403	0.220	0.094	1.723
	Became unemployed or Furloughed due to COVID-19	**2.268**	0.024	1.115	4.614
**Spain N = 1023**	From unemployment to NSE	0.884	0.716	0.454	1.720
	From one NSE to another NSE	1.503	0.101	0.923	2.446
	From SE to NSE	1.942	0.100	0.880	4.284
	Became unemployed or Furloughed due to COVID-19	1.352	0.249	0.810	2.255
**Chile N = 713**	From unemployment to NSE	1.049	0.910	0.457	2.408
	From one NSE to another NSE	1.313	0.465	0.633	2.721
	From SE to NSE	2.091	0.119	0.827	5.287
	Became unemployed or Furloughed due to COVID-19	**2.216**	0.006	1.263	3.889
**U.S. N = 191**	From unemployment to NSE	-			
	From one NSE to another NSE	0.628	0.575	0.124	3.186
	From SE to NSE	3.726	0.092	0.808	17.185
	Became unemployed or Furloughed due to COVID-19	0.760	0.708	0.182	3.177

**Canada**<br>**N = 248**	From unemployment to NSE	1.649	0.326	0.608	4.477
	From one NSE to another NSE	0.621	0.345	0.232	1.667
	From SE to NSE	0.347	0.326	0.042	2.865
	Became unemployed or Furloughed due to COVID-19	1.457	0.443	0.557	3.815

Notes: SE = Standard employment; NSE = non-standard employment. Reference category = Same NSE. The total N sample for each country represents the participants with jointly defined values for the questions about anxiety or depression, employment transitions and, in the case of the adjusted model for age, gender, and education.
